# Stemness Activity Underlying Whole Brain Regeneration in a Basal Chordate

**DOI:** 10.3390/cells11233727

**Published:** 2022-11-22

**Authors:** Tal Gordon, Tal Zaquin, Mark Alec Kowarsky, Yotam Voskoboynik, Noam Hendin, Omri Wurtzel, Federico Caicci, Lucia Manni, Ayelet Voskoboynik, Noa Shenkar

**Affiliations:** 1 School of Zoology, George S. Wise Faculty of Life Sciences, Tel-Aviv University, Tel-Aviv 6997801, Israel; 2 School of Neurobiology, Biochemistry and Biophysics, George S. Wise Faculty of Life Sciences, Tel-Aviv University, Tel-Aviv 6997801, Israel; 3 Department of Marine Biology, The Leon H. Charney School of Marine Sciences, University of Haifa, Haifa 3498838, Israel; 4 Department of Physics, Stanford University, Stanford, CA 94305, USA; 5 Bioinformatics and System Biology, Jacobs School of Engineering, University of California San Diego, San Diego, CA 92093, USA; 6 Department of Biology, University of Padova, 35121 Padova, Italy; 7 Institute for Stem Cell Biology and Regenerative Medicine, and Hopkins Marine Station, Stanford University School of Medicine, Stanford, CA 94305, USA; 8 Chan Zuckerberg Biohub, San Francisco, CA 94158, USA; 9 The Steinhardt Museum of Natural History, Israel National Center for Biodiversity Studies, Tel-Aviv University, Tel-Aviv 6997801, Israel

**Keywords:** stem cells, tunicate, regeneration, central nervous system, transcriptome, gene expression

## Abstract

Understanding how neurons regenerate following injury remains a central challenge in regenerative medicine. Adult mammals have a very limited ability to regenerate new neurons in the central nervous system (CNS). In contrast, the basal chordate *Polycarpa mytiligera* can regenerate its entire CNS within seven days of complete removal. Transcriptome sequencing, cellular labeling, and proliferation in vivo essays revealed that CNS regeneration is mediated by a newly formed neural progeny and the activation of neurodevelopmental pathways that are associated with enhanced stem-cell activity. Analyzing the expression of 239 activated pathways enabled a quantitative understanding of gene-set enrichment patterns at key regeneration stages. The molecular and cellular mechanisms controlling the regenerative ability that this study reveals can be used to develop innovative approaches to enhancing neurogenesis in closely-related chordate species, including humans.

## 1. Introduction

One of the most challenging questions in regenerative medicine is that of how to promote neural regeneration within the adult human brain. In mammals, regeneration is mediated by tissue-specific stem cells capable of self-renewal and multilineage differentiation [1]. While the involvement of neural stem cells (NSC) in neurogenesis of the mammalian brain has been identified [2], the rarity of long term NSCs and the difficulty of monitoring NSCs in vivo, limits our ability to study the mechanisms that direct their activation and differentiation [3,4]. Non-mammalian animals, such as teleost fish, amphibians, and invertebrate species such as tunicates, however, possess expansive regenerative abilities that enable them to replace injured nerve cells, and are used to study the cellular and molecular underpinnings of neural regeneration [5,6,7].

Comparing regeneration mechanisms among a wide array of model systems with varied regenerative capabilities suggests that differences in CNS regeneration ability are due to variations in several key features, including changes in wound-healing responses in neurogenic cell populations and changes in the genetic circuitry of the stem cells that maintains access to embryonic transcriptional programs [8]. Comparing the mouse and zebrafish brain for example, revealed that unlike the mouse, the zebrafish adult CNS contains numerous neurogenic niches with active neuronal progenitors that maintain a life-long ability to produce new neurons in response to injury [9]. In the salamander, CNS regeneration involved the expression of embryonic morphogens to stimulate cell growth, re-patterning, and diversification [8].

In the current study, we developed a new model system to study whole CNS regeneration. By utilizing the solitary tunicate *Polycarpa mytiligera*’s natural ability to regenerate all its body organs and tissues, including a simple brain and CNS, from a small body fragment [6], we have gained insight into neuroregeneration in a species at the base of the chordate evolutionary tree. Tunicates are basal chordates, a sister group of vertebrates, that share structures and cell types considered to be homologous to those in vertebrates [10], and are used as model systems for chordate development and regenerative studies [11,12]. A population of circulatory putative stem cells was suggested to mediate regeneration in this group [13,14,15,16,17,18,19]. Recent transcriptome analyses suggest that these stem cells are tissue specific [10,11]. Following injury, undifferentiated cells accumulate at the regenerating area, forming a proliferation zone capable of producing regenerative tissue [6,16,20,21,22]. A recent study suggests that following CNS ablation, candidate stem cells differentiate into specialized cell types, including neurons [23].

While considerable progress has been made in characterization of the cellular events that lead to CNS regeneration in tunicates, only a few studies have described the expression of morphogens and their transduction pathways that stimulate cell growth, re-patterning, and diversification to regenerate the CNS [11,12,24]. To overcome this gap, we integrated transcriptome sequencing of major CNS regeneration stages with cell labeling and cell proliferation in vivo essays, to characterize the stepwise events and genetic changes that lead to whole CNS regeneration in *P. mytiligera.*

## 2. Materials and Methods

### 2.1. Experimental Design

Our main research aim was to provide a detailed description of CNS regeneration in a new chordate model system. To this end we characterized the morphological, functional, and molecular processes underlying *P. mytiligera* CNS regeneration.

**Figure 1:**
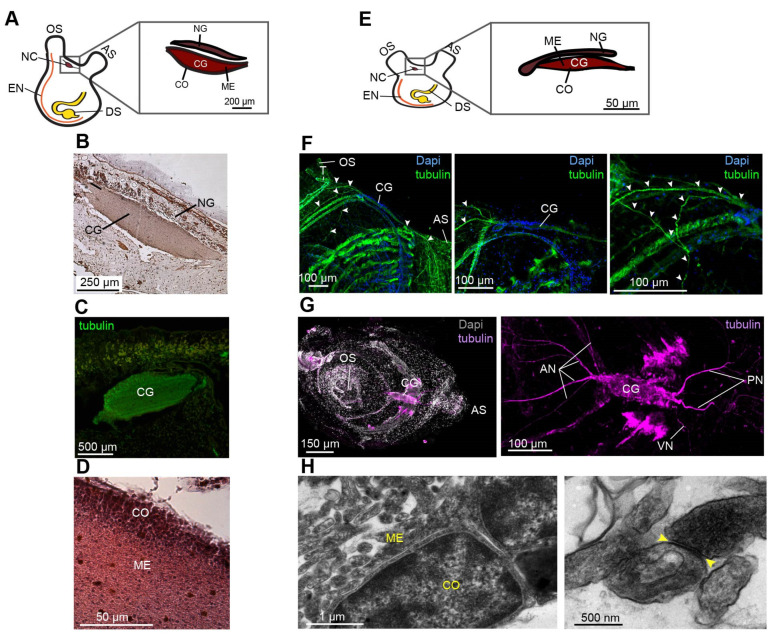
Comparative CNS morphology reveals minimal plasticity at different life stages. (**A**–**D**) *P. mytiligera* CNS morphology during the adult life stage. (**A**) Illustration depicting adult body plan, highlighting the CNS, located between the two siphons, and its components, the cerebral ganglion (CG), medulla (ME), cortex (CO) and neural gland (NG). (**B**,**C**) Representative histological and immunohistochemistry staining of the cerebral ganglion. (**D**) Representative histological section of the cerebral ganglion. (**E**–**H**) *P. mytiligera* CNS morphology during the juvenile life stage. (**E**) Illustration depicting juvenile body plan highlighting the CNS, located between the two siphons and its components: the cerebral ganglion and the neural gland. (**F**) Whole mount immunofluorescence of *P. mytiligera* cerebral ganglion. Maximum intensity projections of confocal stacks. White arrows indicate nerve fibers. (**G**) Whole mount fluorescent in situ hybridization of tubulin showing the cerebral ganglion and associate nerves; anterior nerves (AN), posterior nerve (PN), and visceral nerve (VN). (**H**) Transmission electron microscopy images of *P. mytiligera* neural complex showing the brain structures; medulla (ME) and cortex (CO). Synapse in the medulla is marked with yellow arrows. Atrial siphon (AS), digestive system (DS) endostyle (EN) and oral siphon (OS).

**Figure 2:**
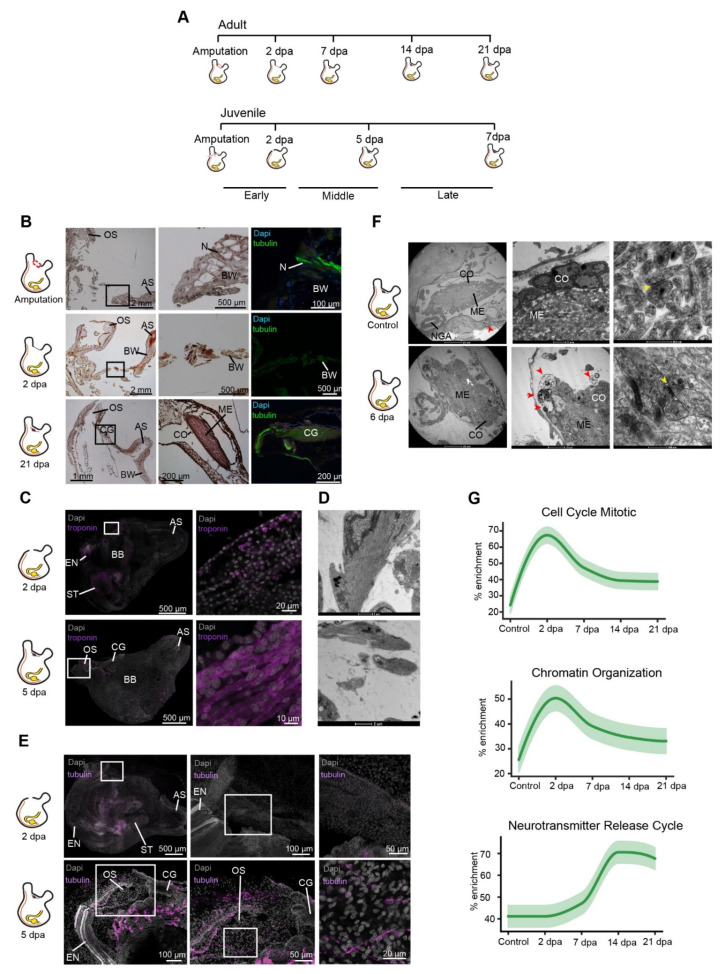
Three distinct anatomical and functional stages characterize CNS regeneration. (**A**) Regeneration timeline showing the difference in regeneration rate between adults and juveniles along the three stages of CNS regeneration. (**B**) Adult animal histological section and immunofluorescent staining (tubulin) showing CNS regeneration process along different time points. Enlargements of the square area appear in the following image in the panel. The CNS is absent following amputation. By 2 dpa we see the beginning of the closing of the open wound followed by complete regeneration of the CNS by 21 dpa. Anterior siphon (AS) body wall (BW) cerebral ganglion (CG), cortex (CO), medulla (ME), nerve fiber (N) neural gland (NG), and oral siphon (OS). (**C**) Whole mount of regenerating animals showing troponin expression in regenerating muscle fibers at 2 and 5 dpa. (**D**) Transmission electron microscopy images of regenerating muscle fibers at 6 dpa. (**E**) Whole mount of regenerating animals showing tubulin expression in regenerating nerves fibers at 2 and 5 dpa. (**F**) Transmission electron microscopy images showing the brain structures and cellular components in control (non-amputated) and 6 dpa animals. Synapse in the medulla is marked with yellow arrows. Immune cells are marked with red arrows. (**G**) Gene enrichment plot of regeneration-associated and CNS related gene sets during CNS regeneration. Light shaded regions indicate the 50% and 99% confidence intervals under a hypergeometric model.

Morphological analysis. We first described the control CNS in juvenile and adult animals in order to better understand this system’s anatomy and to determine the end point of the regeneration process. Next, we monitored the reconstruction of the CNS using various methods, including histology, immunolabeling, and in situ hybridization at different time points following CNS removal (Figure 1). Our findings enabled us to divide the regeneration process into three key stages that reflect similar morphological criteria in both age groups (Figure 2A).

**Figure 3:**
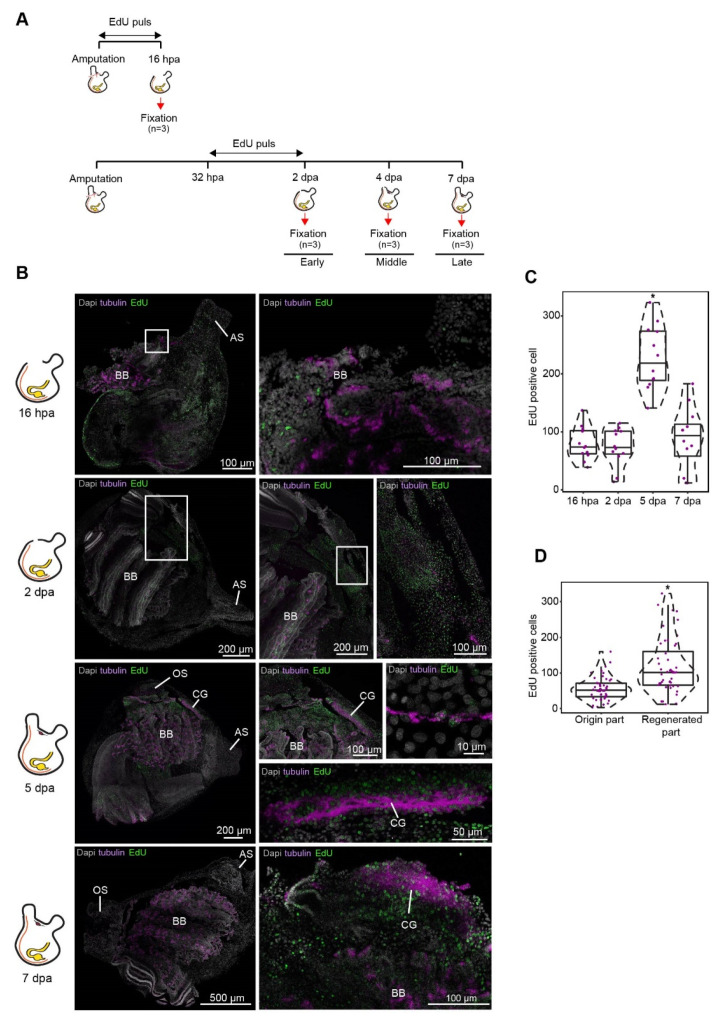
Cell proliferation increase during CNS regeneration. (**A**) Experimental timeline and illustration of experimental paradigm. (**B**) Whole mount EdU staining and tubulin expression during CNS regeneration. All panels are anterior to the top, right view. Whole mount staining of CNS regeneration at 16 h, 2, 5, and 7 dpa showing newly divided EdU positive cells in the regenerated tissue and in regenerating nerve fibers expressing tubulin. Enlargements of the square area appear in the following image in the panel. At 16 hpa the wound is open and the neural complex is absent. Cilia in the exposed branchial basket (BB) are positive for tubulin. At 2 dpa the wound is closed but the neural complex is still absent. EdU positive cells can be seen in the regenerated body wall. At 5 and 7 dpa we see the regenerated cerebral ganglion (CG) composed of tubulin and EdU positive cells. Anterior siphon (AS) and oral siphon (OS). (C and D) Quantification of EdU-positive cells in 100 µm^2^ sections (*n* = 4 sections per animal, *n* = 3 animals per time point). Violin plots display the number of EdU-positive cells in each section. Data are mean ± SE. *P* values determined by Mann–Whitney–Wilcoxon test and are indicated above each boxplot. (**C**) Number of EdU-positive cells in the regenerating tissue along the different time points (*n* = 3 per time point). (**D**) Number of EdU-positive cells in the origin and regenerated tissue along the different time points (*n* = 3 per time point).

Functional analysis. Cell proliferation underlies the formation of new tissues in diverse regenerative organisms, including *P. mytiligera.* Our goals were therefore to better characterize the identity of post-amputation dividing cells and study their involvement in nerve regeneration. First, we sought to describe the location of the proliferation events and determine whether CNS removal resulted in local or systemic cell proliferation. Next, we sought to monitor the identity of the proliferating cells using the tissue-specific markers alpha-tubulin (nerves and cilia) [25,26,27] and troponin (muscle) [28]. To achieve these goals, we applied the EdU proliferation assays to regenerating juveniles and monitored the location of cells that entered the S-phase following CNS removal. We then analyzed the expression of tubulin and troponin in these dividing cells to determine the regenerative stage at which they acquire their identity (Figure 2 and Figure 3).

**Figure 4:**
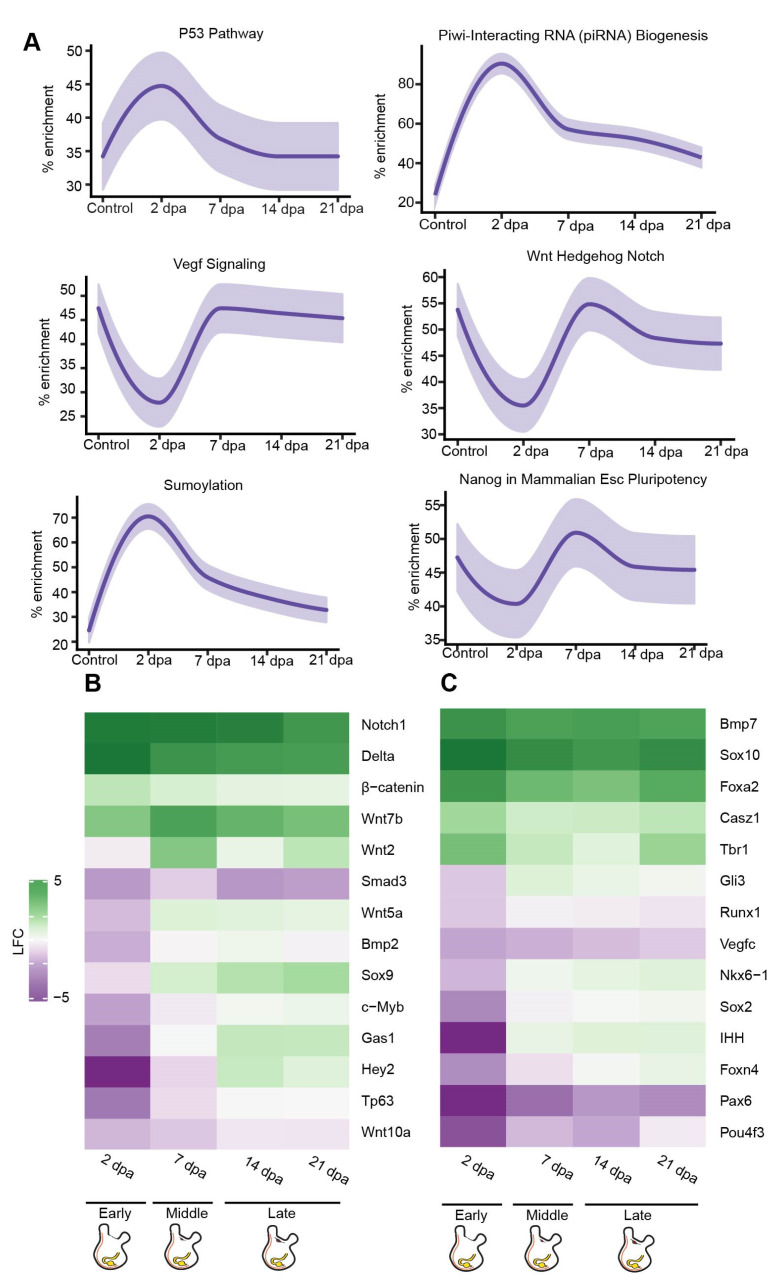
De novo transcriptomic analysis of regenerating CNS reveals expression of stem-cell markers and conserved regeneration associated transcription factors. (**A**) Gene enrichment plot of regeneration-associated genes during CNS regeneration. Light-shaded regions indicate the 50% and 99% confidence intervals under a hypergeometric model. (**B**) Heatmap of log fold change values for selected regeneration related genes significantly differ in comparison to the control. Green indicates a positive fold change (upregulated with respect to uncut CNS), and purple indicates a negative fold change (downregulated with respect to control). (**C**) Heatmap of log fold change values for selected nervous system markers and regeneration related genes significantly differ in comparison to the control. Green indicates a positive fold change (upregulated with respect to uncut CNS), and purple indicates a negative fold change (down-regulated with respect to control).

Molecular analysis. We performed de novo transcriptome analysis using CNS samples of adult animals from the three chosen regeneration stages, in order to analyze the molecular landscape of neural regeneration. As adult stem cells have been shown to mediate whole body regeneration in other tunicates, we hypothesized that CNS regeneration would involve the activation, proliferation, and specialization of a similar population in our highly regenerative model. Focusing on regeneration and stem cell markers, we compared the expression patterns of each regeneration stage with the control and with all other time points in order to identify the enriched pathways and key transcription factors activated or inhibited during CNS regeneration (Figure 2, Figure 4, Figures S3 and S4). This comprehensive analysis provided a valuable insight into the factors and pathways that conduce wound healing and neurogenesis, as revealed by our new chordate model system.

### 2.2. Animal Collection and Culturing

During March-April 2018 *P. mytiligera* adult individuals were collected from the Gulf of Aqaba (Eilat), Red Sea (Israel). The animals were maintained at the Eilat Inter-University Institute (IUI) in aquaria with running seawater for four days of acclimation prior to the onset of the experiments. *P. mytiligera* juveniles were obtained from a breeding culture established in the IUI, as previously described [29].

### 2.3. Regeneration Experiment

For the morphological description of the regeneration process, and selection of representative time points, adults (*n* = 24) of similar size range (4 ± 1 cm, from the tip of the oral siphon to the base of the animal) were anesthetized [30] and dissected. The CNS was removed using a scalpel. Both treated and non-amputated (control) animals (*n* = 4) were maintained in running seawater for the duration of the experiment. Response to touch was used to determine survival. Juveniles of the same age (3 months old, *n* = 24) were dissected, and their CNS was removed. Due to their small size, the oral siphon and the CNS were amputated together to ensure removal of the entire CNS.

### 2.4. Histology and Immunohistochemistry

Regenerating adult animals (*n* = 20) were fixed at different time points along the regeneration process (amputation day, 2-, 7-, 14-, and 21 post-amputation). Following relaxation with menthol, the animals were fixed in 4% formaldehyde in seawater, partly separated from the tunic, and dehydrated with ethanol at increasing concentrations prior to paraffin (Paraplast Plus, Lecia) embedding. Sections (7 μm) were mounted on glass slides, deparaffinized, and stained with standard hematoxylin and eosin solutions.

For immunolabeling, chosen sections were rehydrated in xylene and graded series of ethanol to phosphate-buffered saline (PBS). Juveniles (*n* = 10) were fixed in 4% paraformaldehyde in phosphate-buffered saline (PBS) overnight at 4°C. Fixed juveniles were then washed three times (5 min each) in PBS followed by a 10 min wash in TritonX-100 at room temperature (RT). Samples were blocked in PBT + 3% BSA for 3 h at RT. Antibodies were added directly to the blocking solution overnight at 4°C. To visualize nerves and cilia, we used a mouse monoclonal anti-acetylated tubulin antibody (Sigma T7451) diluted 1:1000 in blocking solution (36, *3*, *7*). Samples were then washed twice for 10 min each in PBT. Secondary antibodies (Alexa Fluor goat anti-mouse IgG 488, ThermoFisher, Waltham, Massachusetts, USA, A-11001) were added at 1:500 dilution to the blocking solution for 3 h at RT. Samples were then washed in PBS three times for 10 min each. Samples were stained with DAPI (Sigma-Aldrich, MO, USA, 1 μg/mL in PBSTx) and mounted. Images were taken using a fluorescent microscope.

### 2.5. Transmission Electron Microscopy (TEM)

To achieve a detailed description of *P. mytilgera* CNS morphology, juveniles (3 months old, *n* = 5) were fixed in 1.5% glutaraldehyde buffered with 0.2 M sodium cacodylate, pH 7.4, plus 1.6% NaCl.

After washing in buffer and post-fixation in 1% OsO4 in 0.2 M cacodylate buffer, the specimens were dehydrated and embedded in epoxy resin (Sigma-Aldrich, MO, USA). Ultra-thin sections (80 nm thick) were stained with uranyl acetate and lead citrate to provide contrast. Photomicrographs were taken with a FEI Tecnai G12 electron microscope operating at 100 kV. Images were captured with a Veleta (Olympus Soft Imaging System) digital camera.

### 2.6. An RNA-Seq Catalog for Central Nervous System Regeneration

To characterize the molecular events that take place during *P. mytiligera* regeneration, and to establish a reference map of CNS regeneration amenable to inter-species comparison, we profiled 12 samples of CNS tissue from adult animals and surveyed gene expression changes. CNS samples (*n* = 3) from untreated adult animals were used to reflect the start and end points of regeneration. Based on these morphological features, pools of regenerating CNS tissues were collected at five time-points: 2 days (*n* = 2), 7 days (*n* = 2), 14 days (*n* = 3), and 21 days (*n* = 2) post-amputation, to provide an informative perspective on the molecular events leading to CNS regeneration.

### 2.7. RNA-Seq, Read Mapping, and Transcriptome Assembly

Samples were prepared at the IUI during September 2017. Fifteen *P. mytiligera* adult individuals (6 ± 1 cm, from the tip of the oral siphon to the base of the animal) were used for neural complex regeneration experiments. Neural complex tissue samples were prepared for RNA extraction (RNAeasy mini kit from Quiagen) at different time points along the regeneration process. Total RNA was extracted following the manufacturer’s protocol. cDNA libraries were then prepared from high-quality samples (RNA integrity number (RIN) > 8) at the Weizmann Institute of Science, Life Sciences Core Facilities, Israel. Barcoded library samples were sequenced on an Illumina NextSeq 500 (2 × 150 bp) at Stanford University, CA, USA.

De novo transcriptome assembly using the Trinity pipeline (https://informatics.fas.harvard.edu/best-practices-for-denovo-transcriptome-assemblywith-trinity.html, accessed on 1 February 2021) was followed (Supp. 1): Adapter sequences and low-quality bases were trimmed using fastp [31]. Sequencing errors were corrected using Rcorrector [32] (v1.0.4) and all reads that were not correctable were discarded. Reads were aligned to the known contaminant *phiX* (Illumina igenome) and known rRNA sequences [33] using Bowtie2 [34] (2.4.1) with the setting very-sensitive-local. Aligned reads were removed. Remaining reads were assembled into transcripts using Trinity [35] (v2.9.1). Assembly quality for each transcript was assessed using TransRate [36] (1.0.3). Only transcripts with p_good >0 score were used for further analysis. TransDecoder [35] (v5.5.0) was used for identifying candidate coding regions within the transcript sequences. Homologous sequences were identified and annotated, based on the candidate coding region transcripts, using BLASTP (NCBI), cut-off 1e-10, to align with (1) the SwissProt protein database; and (2) solely on the *Mus musculus* curated SwissProt database (downloaded on 21 February 2020).

Salmon [37] was used for quantifying gene expression with the following settings: --validateMappings, --numBootstraps 100, --seqBias and –gcBias. Transcript-level abundance were imported using tximport [38] (v1.16.1) to DESeq2 [39] (1.26) for gene-level analysis. Genes with ≤5 supporting reads in ≤2 samples were discarded. The DESeq2 analysis was employed using both the likelihood ratio and Wald tests, to identify all differentially expressed genes between the sequential regeneration stages (FDR < 0.05).

Sets of genes were tested for enrichment of Gene Ontology (GO Biological Process, Molecular Function and Cellular Component) terms. For a set of genes with significant up and down effects, found using the Wald test, an over-representation analysis (ORA) was performed using the enricher function of the clusterProfiler [40] package (v3.16.1) in R with default parameters. A gene set enrichment analysis (GSEA) was also performed on the entire assembly using a scoring based on the log fold change of each gene on its respective time point using the GSEA function of clusterProfiler [40] package (v3.16.1) in R with default parameters. In both cases Significant GO terms were identified with an FDR < 0.05.

The gene ontology terms were obtained from a manually created database based on the SwissProt curated *Mus musculus* GO annotations, using the makeOrgPackage function of AnnotationForge [41]. Pipeline summary is presented in Figure S1.

### 2.8. Identification of Regeneration-Associated Differentially Expressed Genes

The generated dataset was analyzed using two methods:

Identification of differentially expressed genes between each time point and the control homeostatic CNS sample. For each time point, transcript levels were compared between each pool of regenerating fragments and the control sample (Table S1).

Identification of differentially expressed genes between all possible combinations of all time points. The identification of chronologically differentially expressed genes and the formation of binary tables have been described previously [11]. Briefly, we used DEseq2 [39] (*REF*; FDR < 0.05) to identify the differentially expressed genes between all possible combinations of contiguous and individual time points, resulting in a hierarchy of up and down regulated time points for each gene for each age group. For each gene in each experimental group its specific time point hierarchy was then used to score all the possible binary patterns, with each pattern’s score being the number of shared up-and-down regulated time points between it and the hierarchy, while subtracting the number of up-and-down regulated time points for which the pattern and hierarchy disagreed. The pattern with the highest score was used for that gene and regeneration stage pairing. Based on these analyses, a binary gene-time expression matrix for every expressed gene recorded along the time points was produced, with 1 indicating dynamically “high” expression and 0 indicating dynamically “low” expression (Table S2). Using the binary matrix, we identified pathways and Go terms associated with each time point (Figure 2G, Figure 4A and Figure S3B, Table S3).

### 2.9. Gene Enrichment Plots

Gene enrichment plots have been previously described in [11]. Briefly, at each time point, the proportion of genes in a gene set that are active (indicated by a 1 in the gene-time expression binary matrix defined above) is calculated. This gives a value between 0% (no genes in common) and 100% (all genes in the gene set are active at that time). A baseline expectation of the proportion of overlapping genes is calculated using a hypergeometric model that gives the likelihood that the same number of genes as in the selected gene set would be randomly selected from the matrix. In addition, the 68% confidence interval (1 standard deviation) of the proportion of shared genes (‘enrichment’) from the hypergeometric model is calculated, plotted, and presented as a shaded region in the plot. The baseline is then subtracted from the values calculated, with the confidence interval also subtracted, to present the expected range of values and the extent to which the actual enrichment result differs from a null result. If the baseline expectation is greater than the actual enrichment (i.e., the subtracted value will be negative) a value of 0% is used (as a negative percentage is considered meaningless).

### 2.10. Use of Specific Pathway Gene Sets

For the gene enrichment plots the following gene sets were used: For the pathway and go terms identified by GeneAnalytics within our time data, we focused on the pathways of the same names from PathCards, an integrated database of human biological pathways and their annotations. PathCards clustered Human pathways into SuperPaths based on gene content similarity (https://pathcards.genecards.org/, accessed on 21 July 2021). From each of these gene sets we removed any gene that did not have a putative homolog to a known *Polycarpa* gene. Consequently, the percentages within the enrichment plots refer to these curated *Polycarpa* specific gene sets. If a gene name appeared more than once in the *Polycarpa* gene model annotation, all matching *Polycarpa* gene ids were included in the gene set.

### 2.11. Gene Cloning and Transformation

Gene-specific primers were designed from the transcriptome sequence and synthesized by Integrated DNA Technologies (IDT). Their oligonucleotide sequences were as follows: Troponin C 5′GGATTTGACGGAAGAGCAGA3′ and 3′TCATTGCCACGAACTCTTCA5′. Tubulin alpha-1A chain 5′CTGCAGACGAAACCTTGACA3′ and 3′TAAACCGTATCACCGTGCAA5′.

Genes were amplified from *P. mytiligera* cDNAs using gene-specific primers and cloned into pGEM-t vectors using the manufacturer’s protocol (Promega; CAT #A1360). Vectors were transformed into *E. coli* Top10 by the heat-shock method. Briefly, 100 μL of bacteria were mixed with 5 μL of cloned vector, incubated on ice for 30 min, and then subjected to 42°C for 45 sec. The transformed bacteria were then supplemented with 350 μL of SOC medium, and following 1 h of recovery at 37°C, plated on agarose plates containing 1:2000 Ampicillin, 1:200 Isopropylthio-b-D-galactoside (IPTG), and 1:625 5-bromo-4-chloro-3-indolyl-β-D-galactopyranoside (X-gal). Colonies were grown overnight at 37°C, and screened by colony PCR using M13F and M13R primers with the following PCR program: a. 5 min at 95°C; b. 34 cycles of 45 sec at 95°C, 60 sec at 55°C, and 2 h 30 min at 72°C; c. 10 min at 72°C; d. hold at 10°C. Reactions were analyzed by gel-electrophoresis, and correctly sized gene products were grown overnight in Luria Broth media (LB), supplemented with 1:2000 Ampicillin at 37°C. Plasmids were purified from overnight cultures (NucleoSpin Plasmid Miniprep Kit, Macherey-Nagel, Düren, Germany; CAT #740588) cloned gene sequences were sequenced by Sanger sequencing.

### 2.12. Fluorescent In Situ Hybridization

Fluorescence in situ hybridization (FISH) was based on the published protocol [42,43] with some modifications (Appendix A). Essentially, fixed juvenile animals were separated from their tunic and opened along the endostyle. Following bleaching and proteinase K treatments, samples were incubated with DIG/ DNP-labeled riboprobes overnight in hybridization solution, at 60°C. After hybridization, samples were washed twice in: pre-hyb solution, 1:1 pre-hyb-2X SSC, 2X SSC, 0.2X SSC, PBSTx. For the blocking step prior to antibody incubation, 0.5% Roche Western Blocking reagent and 5% inactivated horse serum in 1xPBSTx were used. Samples were then incubated with antibodies (anti-DIG-POD, Fab fragments) overnight at 4°C. Antibody solution was washed with 1xPBSTx. Rhodamine or FITC dyes were used in tyramide development. For peroxidase inactivation, samples were washed with 1% sodium azide solution for 90 min at room temperature. Finally, samples were counterstained with DAPI (Sigma-Aldrich, MO, USA, 1 μg/mL in PBSTx) for 1 h, mounted and photographed using a Zeiss LSM 880 scanning laser confocal microscope.

### 2.13. In Vivo Cell Labeling Experiments

Cell proliferation was detected by incorporating 5-ethynyl-2-deoxyuridine (EdU) into replicating DNA. *P. mytiligera* juveniles (3 months old) were divided into two groups, dissected, and separated from their neural complex and oral siphon using a scalpel (Extended data Figure 3a). One group (*n* = 3) was exposed to a 16 h EdU pulse following amputation and was fixed immediately afterwards. The second group (*n* = 9) was exposed to a 16 h EdU pulse 32 h following amputation and left in seawater to regenerate. Fixation was done at 2-, 5-, and 7 days post-amputation (*n* = 3 per each time point) (Figure 3).

For the pulse experiments animals were incubated with 10 μmol/L EdU (Invitrogen, Carlsbad, CA) in 5 mL of MFSW for 16 h in Petri dishes. Following completion of the labeling, animals were fixed for 12 h in 4% FA, rinsed three times in 1 × phosphate-buffered saline (PBS), and processed for EdU detection using Alexa Fluor azide 488 at room temperature, according to the instructions of the Click-iT EdU Alexa Fluor High Throughput Imaging Assay Kit (Invitrogen). Samples were stained with DAPI (ThermoFisher 33342) (1 μg/mL in PBS) and mounted in VECTASHIELD (Vector Laboratories RK-93952-28) using coverslips.

### 2.14. Statistical Information

All results are expressed as mean ± SE. Statistical analyses were performed using R Studio. Statistical analysis was performed using two-sided Mann–Whitney Wilcoxon test throughout the study. Difference was considered significant as follows: * *p* < 0.05.

## 3. Results and Discussion

To obtain a precise description of the morphological and cellular events underlying *P. mytiligera* CNS regeneration, a detailed description of the CNS anatomy of juvenile and adult animals was acquired using naive animals (Figure 1). Our results revealed a similar morphology in both life stages. The CNS is composed of a single ganglion (brain) [44] and connected by a network of nerves to the different body parts. *P. mytiligera*’s brain consists of an outer cortex and a central neuropil (Figure 1D,H). Nerve cell bodies are present in the cortex, surrounding a medulla where synaptic connections can be seen (Figure 1H). Four main anterior nerves, two posterior nerves, and one ventral nerve exit from the brain (Figure 1F,G). The anterior nerves innervate the incurrent siphon and the anterior body wall, while the posterior nerves innervate the excurrent siphon and the remaining body wall. The ventral visceral nerve extends over the branchial basket toward the digestive tract (Figure 1G). The brain is located adjacent to the neural gland (Figure 1A,B,E), a non-nervous structure that shares with the brain a common embryonic origin [45]. Together, the brain and the dorsal neural gland comprise the neural complex. *P. mytiligera*’s CNS presents a simple brain composed of tubulin-positive nerve cells that interact via synaptic connections, representing a basic model comparable to that in other chordates. Based on these findings, we were able to monitor the CNS regeneration process, differentiate between distinct developmental stages, and determine its endpoint (Figure 2A). Both juvenile and adult animals were able to survive and regenerate following CNS removal. However, the time needed to complete a successful regeneration strikingly differed between the two age groups: young animals regenerated their entire CNS within 7 days, while older animals needed 3 weeks to complete the same process (Figure 2A). To study the cellular and transcriptomic signatures associated with CNS regeneration, we compared CNS samples taken from control animals (to establish a basal state) with CNS collected at diverse time points along the regeneration process. Based on these morphological and transcriptomic results, CNS regeneration was divided into three milestones: early, middle-, and late-stage regeneration (Figure 2A).

To better characterize the cells that had contributed to the regenerated tissues we labeled endogenous dividing cells through the systemic administration of EdU, which incorporated into the DNA during the S-phase of cell division and stained the nucleus of the cells that underwent division within a few hours post-treatment (Figure 3). Fluorescent in situ hybridization of tissue-specific markers was then used to track these newly dividing cells and identify the ones that had differentiated into neurons (Figure 2E and Figure 3B) [46]. Our results indicate a possible contribution of the undifferentiated progenitor cells that enter the S-phase cellular state during the early regeneration stages, to the formation of the regenerated CNS. Following amputation of the CNS, only the isolated tubulin- positive nerve fibers in the surrounding tissue remained (Figure 2B). At the initial stage post-amputation epidermal tissue started to regenerate and close the open wound (Figure 2B). This tissue was composed of regenerating troponin-positive muscle fibers (Figure 2C,D), while nerve fibers had not yet began to re-innervate the tissue (Figure 2E). During the first 16 h post-amputation (hpa) no significant difference in the number of EdU positive cells was found between non-regenerated and regenerated areas. At 48 hpa a higher number of proliferating cells were located specifically in the regenerated area, indicating a local increase in proliferation rate. At this time point, these dividing cells did not express CNS specific markers (Figure 2E and Figure 3B).

During the mid-regeneration stage, the wound became fully healed, and the CNS regenerated, with newly formed nerve fibers exiting from the brain and continuing along the regenerating tissue to the siphons (Figure 2E). This stage was also characterized by the highest number of EdU-positive cells. Some of these cells were found embedded in the regenerated brain and nerve fibers and were tubulin-positive, indicating their neuronal identity (Figure 3B). This finding indicated a recent differentiation event, as these neural progenies had acquired their identity as nerve cells at this time point, following their proliferation during the early regeneration stage.

At the late regeneration stage, the de novo brain morphological structures and cellular distribution pattern resembled those of the control (Figure 2B). TEM analyses verified that the regenerated brain contained a cortex and a medulla with visible synapses. However, the brain morphology was still not completely identical to the control brain at this point, as the cortex layer was not yet well organized into distinct layers (Figure 2F). In addition, we observed several immune cells embedded in the cortex layer (Figure 2F), implying a possible contribution of the immune system to the regeneration process [47].

To characterize the molecular signature of neural regeneration we performed the first transcriptome assembly of a solitary chordate CNS regeneration (Figure S1).

The generated dataset was then analyzed using over-representation (ORA) and functional enrichment (GSEA), to identify the key pathways that had been enriched during the CNS regeneration in comparison to the control CNS (non-amputated brain) (Figure S1). Moreover, by combining the DEseq2 [39] and bioinformatic tools that we had developed (all vs. all analysis [11]), we were able to construct a detailed gene expression atlas of the differentially expressed gene sets for each stage of the regeneration process (Table S2). Our analyses revealed the dynamic expression of 239 pathways, demonstrating significant changes in gene expression along the regeneration timeline.

The early regeneration stage was characterized by activation of cell proliferation and chromatin organization pathways (Figure 2G), consistent with the results of our EdU assay. The main upregulated GO terms comprised cell signaling, nerve proliferation, and injury response pathways, reflecting the animal’s fast response to the amputation and damaged tissues (Figure S2). Interestingly, this stage was enriched in several stem-cell-related pathways (Figure 4A), including the p53 pathway, a stress response pathway required for neurite outgrowth as well as for axonal proliferation and regeneration [48]. In the mouse brain, p53 is involved in maintaining regenerative ability by regulating the proliferation of stem and progenitor cells [49]. p53 has been shown to play several roles, including apoptosis, cell cycle arrest, and proliferation, depending on the different stages of the regeneration process [50]. In *P. mytiligera,* activation of this pathway occurs at the early regeneration stage and is inhibited at the later stages. These findings indicate a negative regulation of neural stem cell self-renewal and an investment in specialization processes at the early time point, followed by stem cell maintenance as the CNS undergoes reconstruction [51,52]. p53 transcriptional activities are primarily regulated through post-translational modifications, including sumoylation [53]. The sumoylation pathway was also enriched during the early stage of CNS regeneration (Figure 4A). This pathway has a role in neural crest development [54] and stem-cell proliferation [55], and was shown to increase in mice following nerve lesion [56]. Another post-transcriptional regulatory pathway that was highly enriched following *P. mytiligera*’s CNS ablation was the Piwi-piRNA pathway (Figure 4A). This pathway is involved in stem cell maintenance in diverse organisms [57], and was suggested to have an inhibitory role in neuron or Schwann cell responses during peripheral nerve injury in both nematodes and rodents [58].

As regeneration progressed, dynamic changes in the enriched pathways could be observed. Upregulation processes associated with differentiation and tissue formation were expressed, as well as the enrichment of extracellular matrix organization processes (Figure S2). Enrichment of Vegf signaling indicates a revascularization process, an essential process during regeneration and consistent with the morphological description (Figure 4A). At this stage the pathways associated with stem-cell activity that had been detected at 2 dpa were replaced by a different set of stem-cell associated pathways, including Notch, Hedgehog, and Wnt (Figure 4A), indicating a possible shift from stem cell proliferation and differentiation to stem, cell self-renewal and maintenance [50,59,60,61,62]. Closer observation of the gene sets composing these pathways revealed potential key regulators of CNS regeneration, including *wnt7b, wnt5a,* β-catenin, low-density lipoprotein receptor-related protein 5 (*lrp5*), and *lrp6*, all of which demonstrated a dynamic expression pattern along the regeneration process (Figure 4B,C, Figures S3 and S4). Components of the Notch signaling pathway included upregulation of the receptor protein gene *notch-1* and its ligand, *delta,* down-regulation of the signal mediator *rbpj*, and dynamic expression of a downstream transcriptional target *hey-2* (Figure 4B,C, Figures S3 and S4).

The middle and late regeneration stages demonstrated an enrichment of the signaling pathways associated with tumor necrosis receptor factor 1 (TNFR1), fibroblast growth factor receptor (FGFR), and nerve growth factor (NGF) (Figure S3B). These pathways play key roles in controlling the normal inflammatory and wound response to muscle and neuronal damage, necessary to achieve regeneration [47,63].

The late stage de novo regenerated brain revealed an upregulation of GO terms related to extracellular matrix secretion and axis specification (Figure S2B), implying a continuous process of tissue assembly and specialization. Additional support for such a shift came from the upregulation of voltage-gated ion channel genes, including *kcnh8* and *cacnb2* (Figure S3A), and the enrichment of gene sets associated with the Nanos pathway (Figure S4B) and neurotransmitter release cycle (Figure 2G), reflecting morphogenesis processes and the establishment of functional synapses [64].

Next, we sought to identify key regulatory factors that are known to be involved in CNS regeneration in other model organism, that were differentially expressed during regeneration in *P. mytiligera*, and to determine precisely when their expression was enhanced or inhibited along the regeneration process in relation to the control (Figure 4B,C, Figures S3 and S4). Our results revealed a dynamic expression of transcription factors related to neuronal differentiation and neural cell fate specification (Figure 4B,C, Figures S3 and S4). Among this gene list, *sox10*, *tbr1*, and *pax-6* were differentially expressed following CNS removal. Fluctuation in expression patterns of these markers has been previously shown to mediate CNS regeneration in other chordates [65,66]. These findings indicate a possible conserved role of these factors during nerve regeneration in *P. mytiligera*, as both *sox10* and *tbr1* were upregulated throughout the entire process, while *pax-6* was consistently down-regulated.

The nervous system development genes, *foxa2* and *six1*, were also upregulated during *P. mytiligera* CNS regeneration (Figure 4B,C, Figures S3 and S4). A similar trend in expression patterns of these factors underlies regeneration in mice, where *foxa2* is required for the specification and differentiation steps of neurons at progressively higher doses [67] while *Six1* expression is required for the proliferation of neuroblast progenitors [68]. Interestingly, the neurogenic transcription factors *c-myb* and *sox2* were upregulated during the late stage of CNS regeneration. The universal neural stem cell marker *Sox2* is involved in the proliferation and maintenance of neural stem cells as well as in neurogenesis [69]. The hematopoietic transcription factor and proto-oncogene *c-myb* regulates neural progenitor proliferation and has an important regulatory role in the neurogenic niche of the adult mouse brain. The expression of these neural stemness markers following CNS removal strongly suggests the involvement of stem cells during regeneration in *P. mytiligera*.

**Figure 5:**
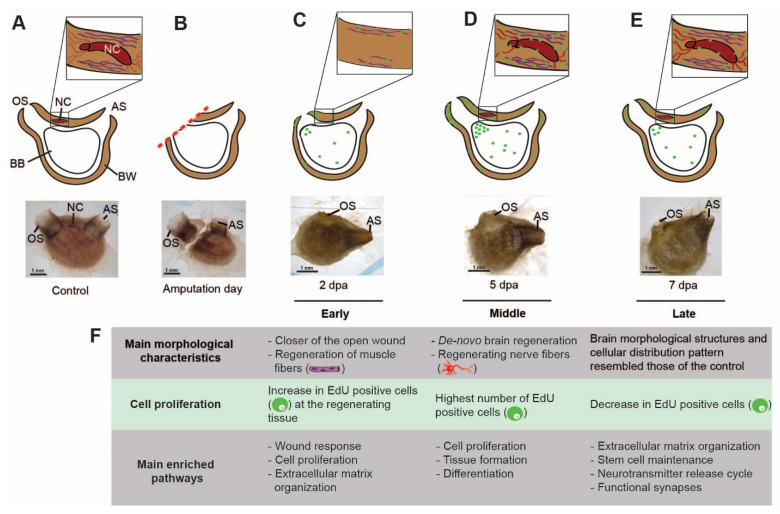
The cellular and transcriptome blueprint of CNS regeneration. (**A**–**E**) Summary of the main cellular events leading to CNS regeneration (green—EdU positive cell, red—nerve fibers, and purple—muscle fibers). (**A**) Illustration and in vivo image showing a control (pre-amputation) animal. The cerebral ganglion (CG) is embedded in the body wall (BW). Nerve fibers connecting the brain with the surrounding tissue. Muscle fibers embedded in the body wall, surrounding the brain. Atrial siphon (AS), branchial basket (BB), and oral siphon (OS). (**B**) Illustration and in vivo image showing an animal immediately following amputation. (**C**) Illustration and in vivo image showing an animal at early stage of regeneration (2 dpa). The open wound is closed by epidermal tissue composed of newly regenerating muscle fibers. The brain and associated nerves are absent. Higher levels of EdU positive cell embedded in the regenerating tissue in comparison to non-regenerating tissue. (**D**) Illustration and in vivo image showing an animal at middle stage of regeneration (5 dpa). The open wound is closed by epidermal tissue composed of regenerating muscle fibers. Nerves fibers appear at this stage and are connected to the regenerated brain. At this point we see the highest level of EdU positive cells embedded in the regenerating tissue. (**E**) Illustration and in vivo image showing animal at the late regeneration stage (7 dpa). At this point we see a decrease in the level of EdU-positive cells embedded in the regenerating tissue, albeit still higher than the level found in non-regenerating tissue. (**F**) Summary of the main morphological and molecular processes involved in CNS regeneration.

Our study presents gene sets and pathways known to be associated with neural regeneration and stem cells and describes the exact time point in which their expression was upregulated or down-regulated (Table S3). Our results indicate that CNS regeneration in *P. mytiligera* shares basic functional stages with other regenerative animals. These include an increase in cell proliferation and reorganization process at early stages of regeneration, followed by the activation of specialization and morphogenesis processes at late regenerative stages (Figure 5). While we chose to focus on conserved pathways, known to mediate regenerative response in other organisms, our analyses revealed novel relations between the expression dynamics of some of these pathways that were unique to our model. These include the high expression of P53 and piRNA pathways preceding the activation of Notch, Wnt, and Nanos pathways at early stages of the CNS regeneration. As a member of an evolutionary clade considered to be the closest living invertebrate relative of vertebrates, our CNS regeneration atlas may well constitute a fundamental resource in understanding how evolution has shaped CNS regeneration processes across phylogeny, and in obtaining mechanistic insight into this complex process.

Utilizing a variety of model organisms contributes greatly to our understanding of regenerative biology, and establishing new models bears the potential to uncover new insight into this complex process. However, working with non-model organisms has its limitations and challenges [70,71]. One of the main challenges of the current study was the low number of available specimens as well as availability of experimental tools and protocols. To overcome these challenges, we established new protocols (Appendix A) and divided our pool of accessible animals into several selected experiments. Juvenile animals were used for FISH and EdU experiments, where their small size and transparency represent a significant advantage, while adult animals were used for RNA extraction and gene expression analysis as their large body size enables accurate removal of the CNS. Another limitation of working with a non-model is the need to establish a broad and somewhat basic foundation for future research. Our regeneration atlas has revealed the main pathways and genes that are activated or inhibited throughout the entire CNS regeneration process in an invertebrate chordate species, and suggests the involvement of candidate stem cells in this process. These findings will pave the way for future studies aimed at functionally isolating neural stem cells in adult tissues, and testing the involvement of key genes in directing their differentiation to form de novo whole CNS.

## 4. Conclusions

As the closest living relative of vertebrates, the tunicates serve a critical role in understanding developmental processes that are comparable to those of vertebrates. Our study has revealed a detailed road map of complete CNS regeneration, in which the activation and inhibition of candidate neurogenesis factors are documented. CNS removal initiates the proliferation of progenitor cells that differentiate into neurons and trigger the expression of known stem-cell transcriptional regulators, including *sox2*, *hes1*, and *c-myb*. As recruiting endogenous and exogenous NSC for CNS regeneration has become one of the main goals of regenerative medicine [72,73], the identification of conserved NSC-activating genes in this simple model system offers a promising approach for developing therapeutic applications in vivo.

## Data Availability

The datasets generated for this study can be found in the NCBI Sequence Read Archive under accession number PRJNA773979.

## Supplementary Materials

The following supporting information can be downloaded at: https://www.mdpi.com/article/10.3390/cells11233727/s1, Figure S1: *P. mytiligera* de novo transcriptome assembly summary; Figure S2: Gene ontology categories enriched at the different stages of CNS regeneration; Figure S3: Expression profile for genes involved in nerve regenerative functions; Figure S4: Expression profile for genes involved in regenerative functions; Table S1: Complete dataset of significantly differentially expressed genes (FDR < 0.05) and transcript counts at different time points of CNS regeneration (logFC and *p*-values); Table S2: Binary gene lists expressed; Table S3: Pathway enrichment expression along *P. mytiligera* CNS regeneration stages based on GeneAnalytics analysis.

## Appendix A

Whole-mount fluorescent in situ hybridization staining of the solitary tunicate *Polycarpa mytiligera*

Day 1

Anesthetize animals with menthol crystals in seawater until siphons open and become unresponsive.

Incubate animals in fixative solution overnight at 4°C.

Incubate in 50% methanol in PBST for 10 min at room temperature.

Incubate in 100% methanol for 10 min (2 x) at room temperature or at −20°C for long-term storage.

Incubate in 100% methanol.

Incubate in 75% methanol in PBST for 10 min at room temperature.

Incubate in 50% methanol in PBST for 10 min at room temperature.

Incubate in 25% methanol in PBST for 10 min at room temperature.

Incubate in PBST for 10 min (2 x) at room temperature.

Incubate in bleaching solution without nutation for 30 min to two hours at room temperature until fully bleached. Use light table for better results.

Incubate in PBST for 10 min (2 x) at room temperature.

Treat samples with proteinase K for 15 min at room temperature without nutation.

Rinse samples with PBST.

Fix samples with 4% formaldehyde in PBST for 20 min at room temperature without nutation.

Incubate in PBST for 10 min at room temperature.

Incubate in 0.5% Triton X-100 for 15 min at room temperature.

Transfer samples into baskets in a 24-well tissue culture plate.

Move baskets into 50% pre-hybridization buffer in PBST and shake for 15 min at room temperature.

Incubate baskets in pre-hybridization buffer for at least 4 h at 58–65°C without nutation. Keep the plate in dark from this step on.

Prepare riboprobes mix in hybridization buffer (1:800).

Incubate the mix for 5 min at 72°C and add it to the 24-well plate.

Incubate at 58°C for 15 min.

Hybridize samples with riboprobes mix overnight at 58°C without nutation (do not incubate for more than 16 h).

Day 2

Incubate baskets in 67% pre-hybridization buffer in 2X SSCT for 15 min at 58°C without nutation.

Incubate baskets in 33% pre-hybridization buffer in 2X SSCT for 15 min at 58°C without nutation.

Incubate baskets in 2X SSCT for 15 min at 58°C without nutation.

Incubate baskets in 0.2X SSCT for 30 min at 58°C without nutation.

Incubate baskets in 0.2X SSCT for 45 min at 58°C without nutation.

Incubate baskets in 67% 0.2X SSCT in PBST for 5 min at room temperature.

Incubate baskets in 33% 0.2X SSCT in PBST for 5 min at room temperature.

Incubate baskets in PBST for 5 min (2 x) at room temperature.

Incubate baskets in Blocking solution for 4 h at room temperature.

Incubate baskets with antibody solution overnight at 4°C with motion.

Day 3

Wash samples in PBST for 20 min (8 x) at room temperature.

Incubate in TSA for 10 min at room temperature.

Incubate in Tyramide solution 1:1000 for 20 min at room temperature.

Add 10 ul of 30% H202 (1:300) to each well for 45 min.

Incubate in PBST for 10 min (6 x) at room temperature.

Incubate in 1 µg/mL DAPI in PBS for one hour at room temperature to stain nuclei.

Incubate in PBST for 10 min (3 x) at room temperature.

Flat-mount samples.

Recipes

PBST buffer: 1X PBS pH 7.4, 1:1000 Triton X-100

Fixative solution: 4% Formaldehyde in PBST.

Bleaching solution: 5% Formamide, SSCx0.5, 1.2% H2O2.

Proteinase K solution: 10 µg/mL proteinase K in PBST.

Pre-hybridization buffer- 50% Formamide SSCx5, 2.5 mg/mL yeast RNA, 1% Tween-20, 1x Denhardt’s solution.

Hybridization buffer: 50% Formamide SSCx5, 2.5 mg/mL yeast RNA, 1% Tween-20, 1x Denhardt’s solution, 5% Dextran sulfate.

Blocking solution: 5% heat inactivated horse serum, 0.5% RWBR in TNT.

Antibody solution: anti-DIG HRP-conjugated antibody (1:500) in Blocking solution.

TNT buffer: 0.1 M Tris pH 7.5, 0.15 M NaCl, 0.3% Triton X-100

TSA buffer: 2M NaCl, 0.1M Boric acid, PH 8.5

Tyramide solution: 0.3% H2O2 (1:100), 4IPBA (1:1000), Rhodamine (1:1000) in TNT buffer.

Non-Standard Reagents

Formaldehyde 37% (Sigma-Aldrich, 1040031000)

Formamide deionized (Mercury, S4117)

RNA from yeast (Roche, 10109223001)

RWBR- Western Blocking Reagent (Sigma, 11921673001)

Donor Horse Serum, Heat Inactivated (Biological Industries, 04-124-1A)

4IPBA- 4-Iodophenylboronic acid (Sigma, 471933)

Anti-Digoxigenin-Rhodamine, Fab fragments (Sigma-Aldrich, 11207750910)

30% H_2_O_2_ (Sigma-Aldrich, H1009-100ML)

Proteinase K Solution, RNA grade (Invitrogen, 100005393)

Rhodamine (Thermo Fisher Scientific, TS-46406)

Dextran sulfate (Sigma, D8906)
