# Supplementary material for: Stemness Activity Underlying Whole Brain Regeneration in a Basal Chordate
Source: Cells. 2022 Nov 22;11(23):3727. doi: 10.3390/cells11233727 (PMC9738451; doi:10.3390/cells11233727)
Supplement: Supplementary file 1 [file cells-11-03727-s001.zip › Cells_Supplementary_figures_Gordon_revised.pdf]

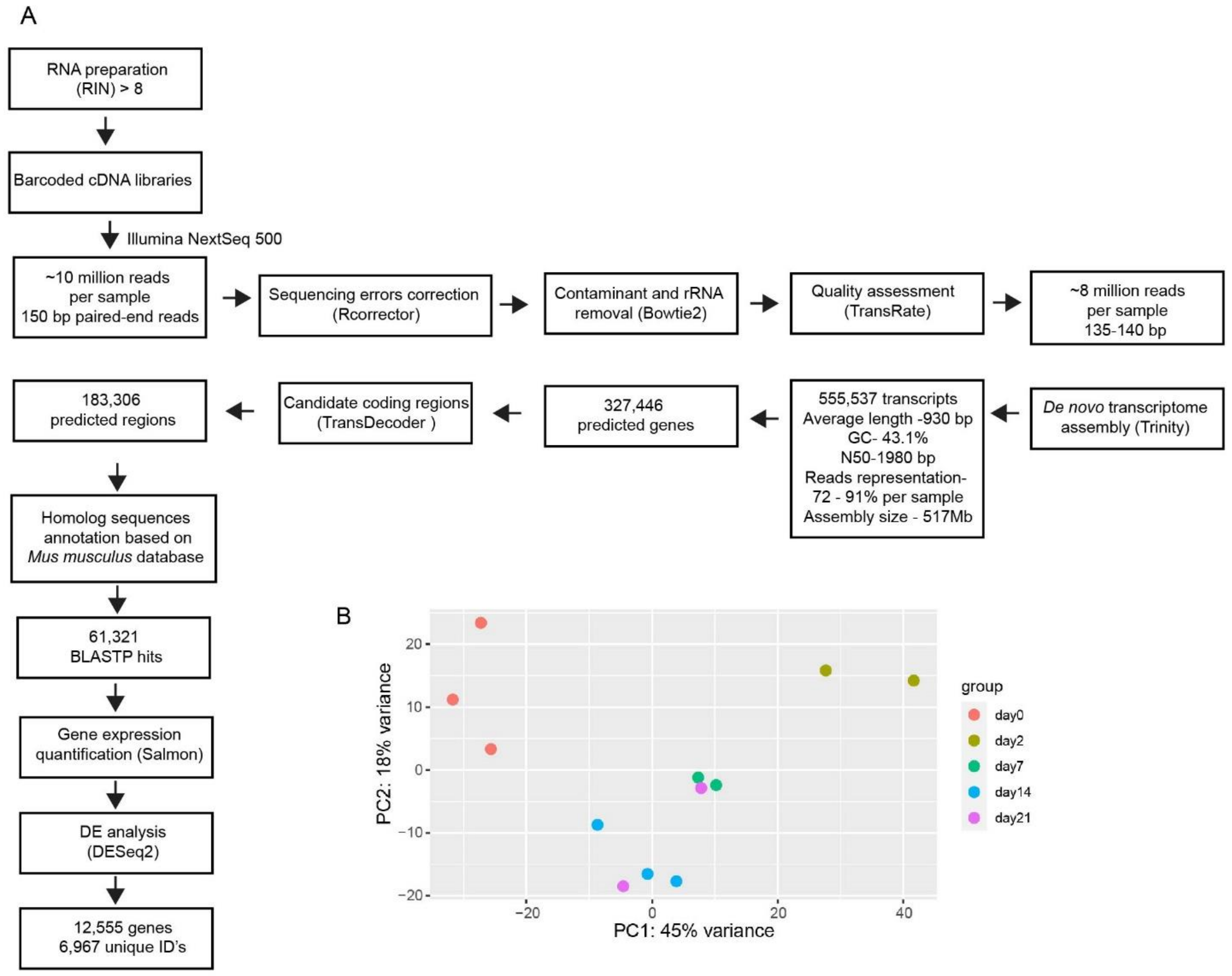

Figure S1. ***P. mytiligera de-novo*** **transcriptome assembly summary.**
(**A**) Pipeline for *P. mytiligera* RNAseq and *de-novo* assembly process. (**B**) Principal component analysis (PCA). RNA-Seq data sets from control and different time points of CNS regeneration show separation between the CNS control sample and the regenerating samples, and between the early stage of regeneration (2 dpa) and late stages (7, 14 and 21 dpa), which are grouped together.

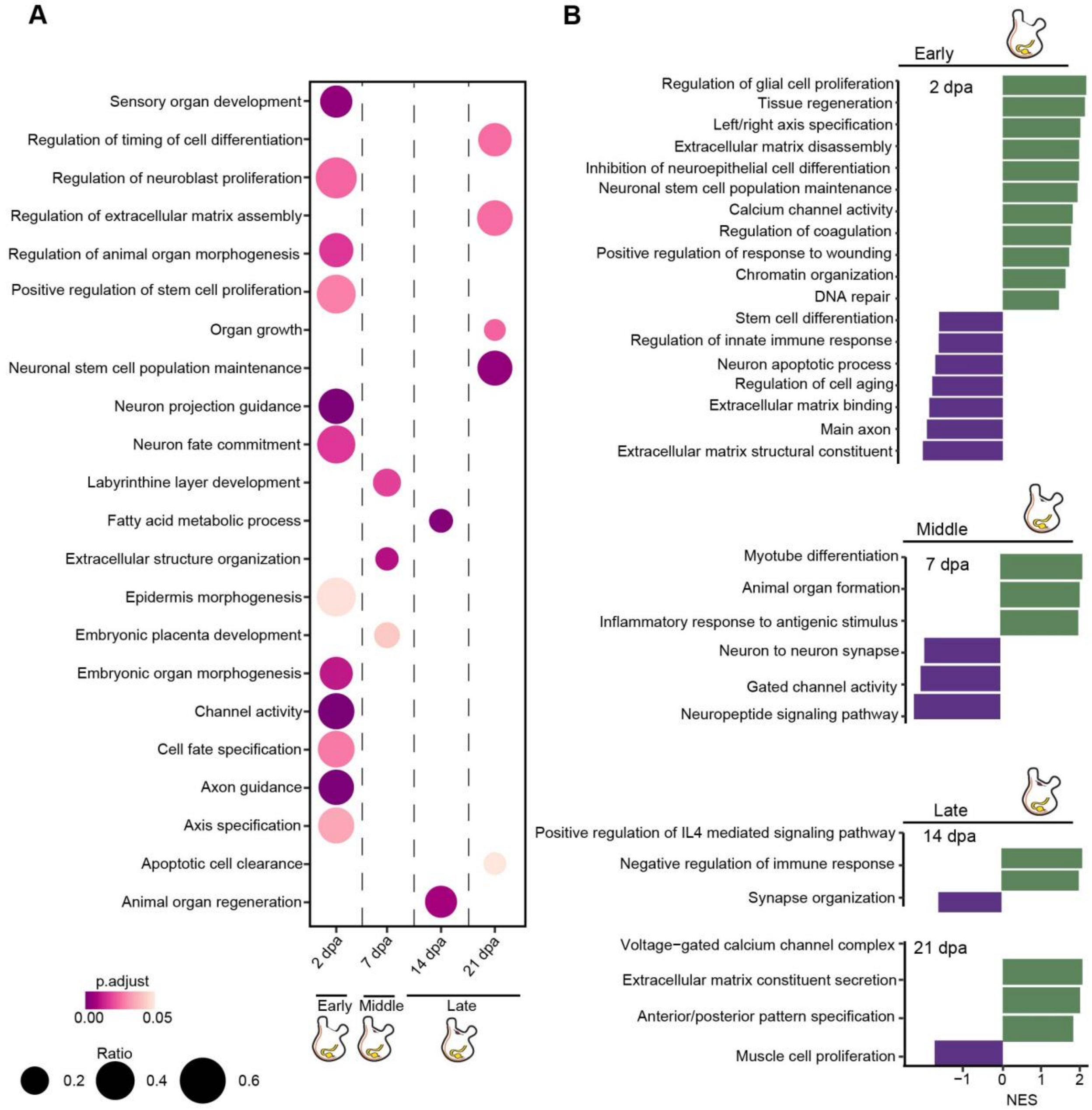

Figure S2. Gene ontology categories enriched at the different stages of CNS regeneration.
(**A**) Gene ontology (GO) term enrichments for each of the time points. The enrichment of each GO term is indicated by a circle, where the area corresponding to the fraction of genes annotated with that term is present in the cluster, and the color of the circle corresponds to the adjusted p-value of term enrichment (ORA, FDR≤0.05). (**B**) Gene sets are plotted relative to normalized enrichment scores (NES). Categories with negative or positive NES are down- or upregulated, respectively (GSEA, FDR <0.5).

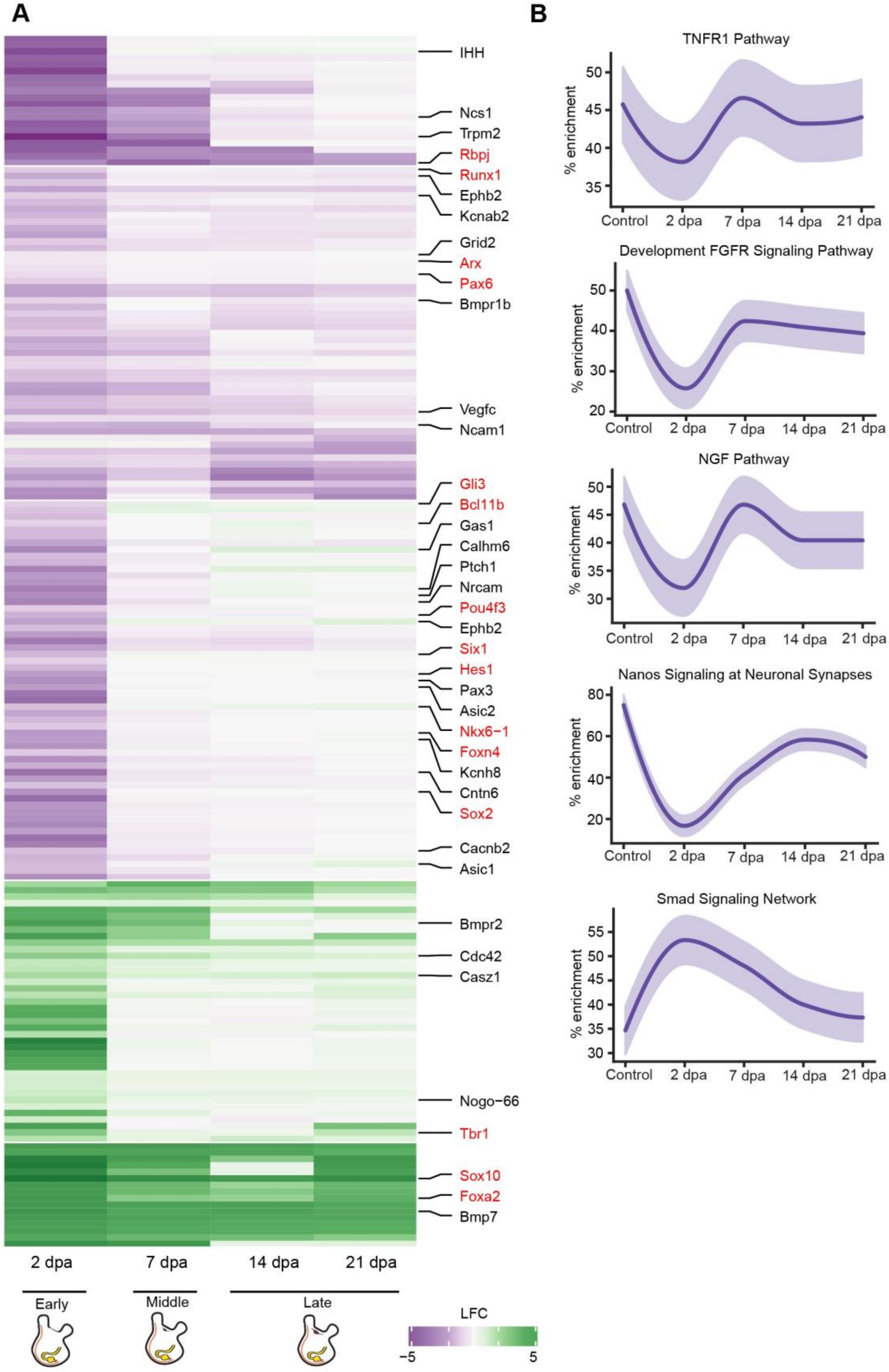
A
B
IHH
Ncs1
Trpm2
Rbpj
Runx1
Ephb2
Kcnab2
Grid2
Arx
Pax6
Bmpr1b
Vegfc
Ncam1
Gli3
Bcl11b
Gas1
Calhm6
Ptch1
Nrcam
Pou4f3
Ephb2
Six1
Hes1
Pax3
Asic2
Nkx6−1
Foxn4
Kcnh8
Cntn6
Sox2
Cacnb2
Asic1
Bmpr2
Cdc42
Casz1
Nogo−66
Tbr1
Sox10
Foxa2
Bmp7
2 dpa
7 dpa
14 dpa
21 dpa
Early
Middle
Late
LFC
−5
5
TNFR1 Pathway
Development FGFR Signaling Pathway
NGF Pathway
Nanos Signaling at Neuronal Synapses
Smad Signaling Network
% enrichment
Control
2 dpa
7 dpa
14 dpa
21 dpa

Figure S3. Expression profile for genes involved in nerve regenerative functions.
(**A**) Heatmap depicts log fold change values for significantly expressed genes (rows) over the sampled regeneration time points. Green indicates a positive fold change (upregulated with respect to uncut CNS), and purple indicates a negative fold change (downregulated with respect to control). Genes of interest appear on the right. Regulatory factors are colored red.
(**B**) Gene enrichment plot of regeneration-associated genes during CNS regeneration. Light-shaded regions indicate the 50% and 99% confidence intervals under a hypergeometric model.

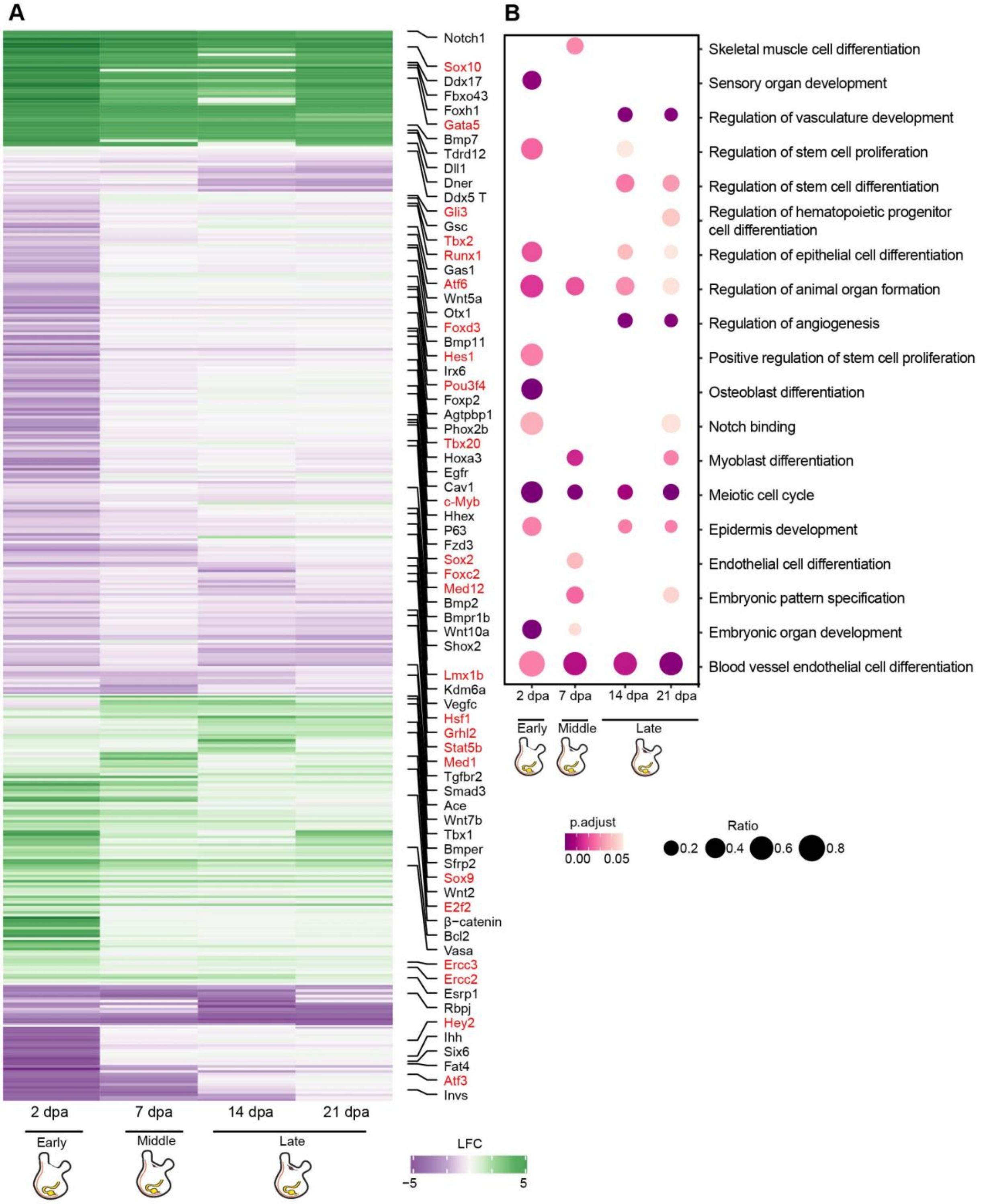

Figure S4. Expression profile for genes involved in regenerative functions.
(**A**) Heatmap depicts log fold change values for significantly expressed genes (rows) over the sampled regeneration time points. Green indicates a positive fold change (upregulated with respect to uncut CNS), and purple indicates a negative fold change (downregulated with respect to control). Genes of interest

appear on the right. Transcription factors are colored red. (**B**) Regeneration related gene ontology (GO) term enrichments for each of the time points (ORA, FDR≤0.05).
