# Supplementary figures and images for: Stemness Activity Underlying Whole Brain Regeneration in a Basal Chordate
Source: Cells. 2022 Nov 22;11(23):3727. doi: 10.3390/cells11233727 (PMC9738451; doi:10.3390/cells11233727)

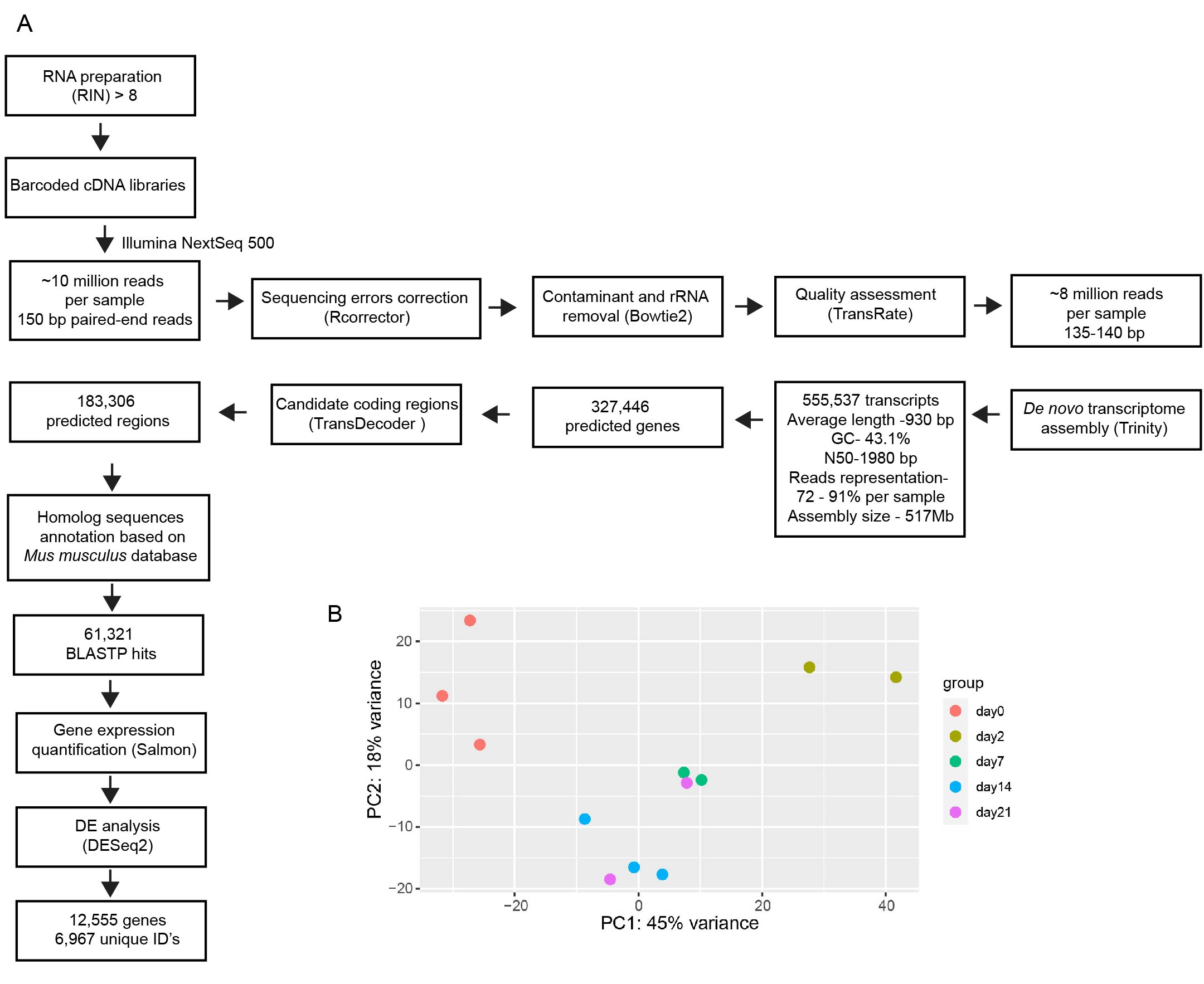

Supplement: Supplementary file 1 [file cells-11-03727-s001.zip › Fig S1.jpg]

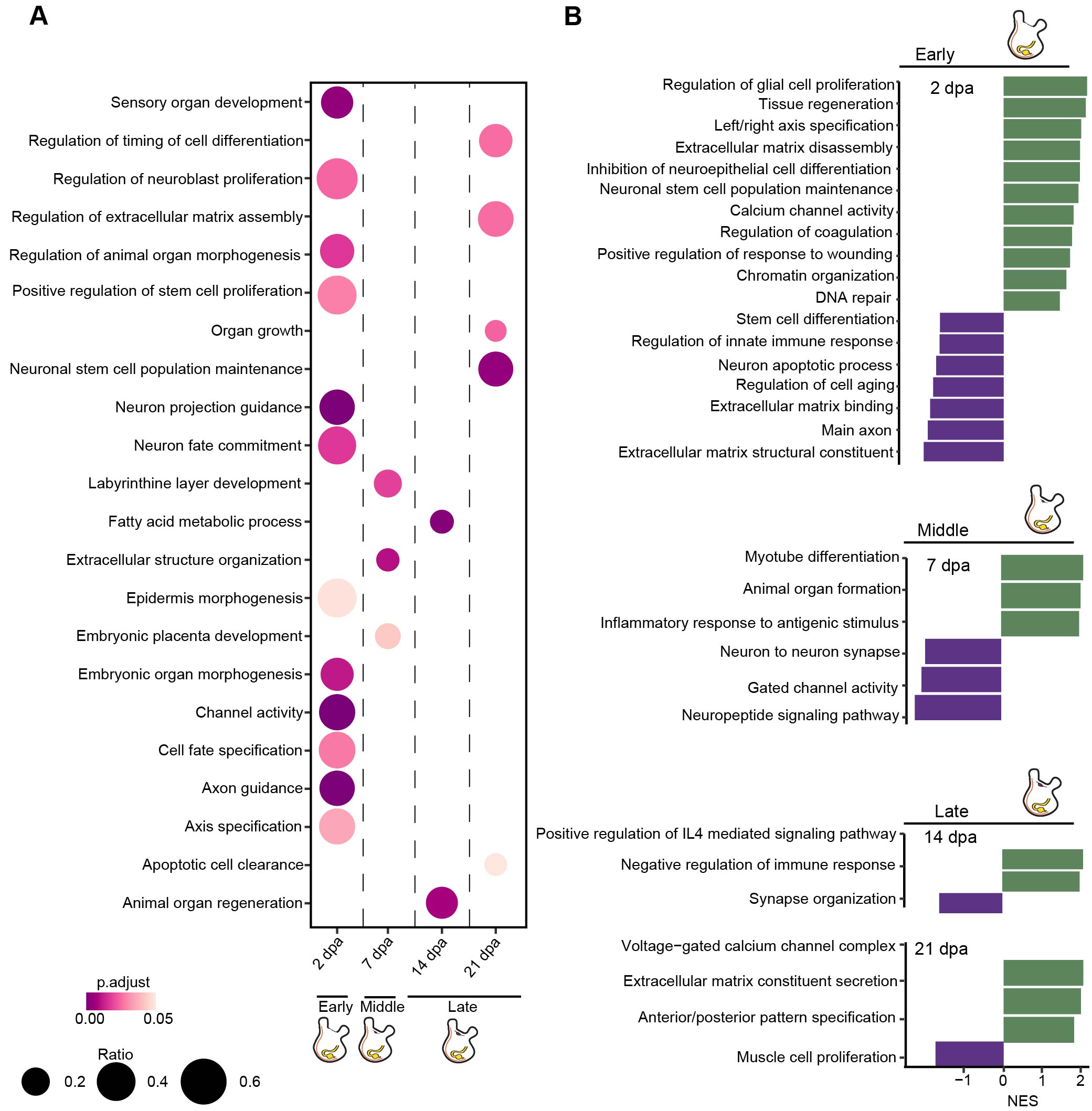

Supplement: Supplementary file 1 [file cells-11-03727-s001.zip › Fig S2.jpg]

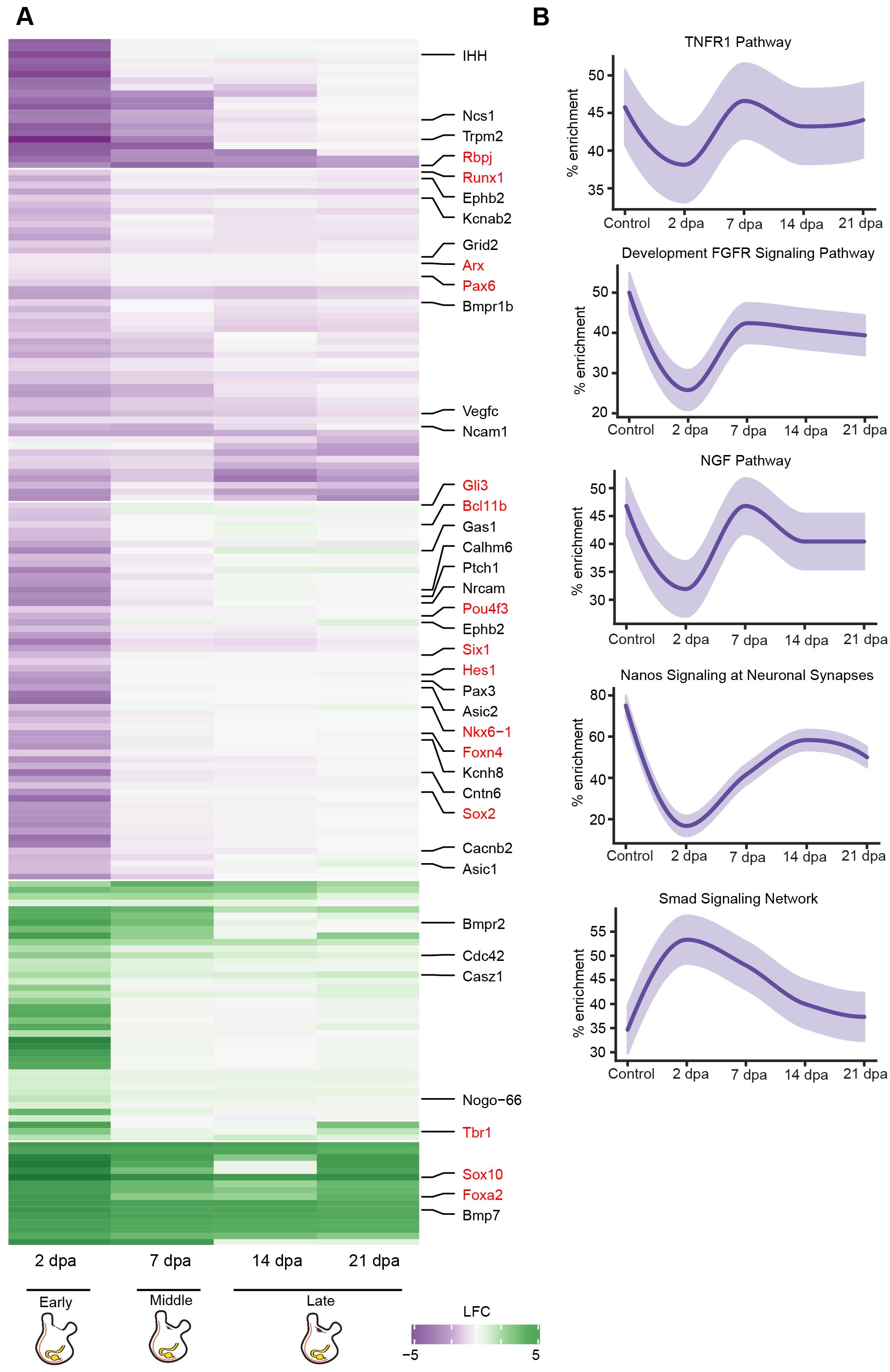

Supplement: Supplementary file 1 [file cells-11-03727-s001.zip › Fig S3.jpg]

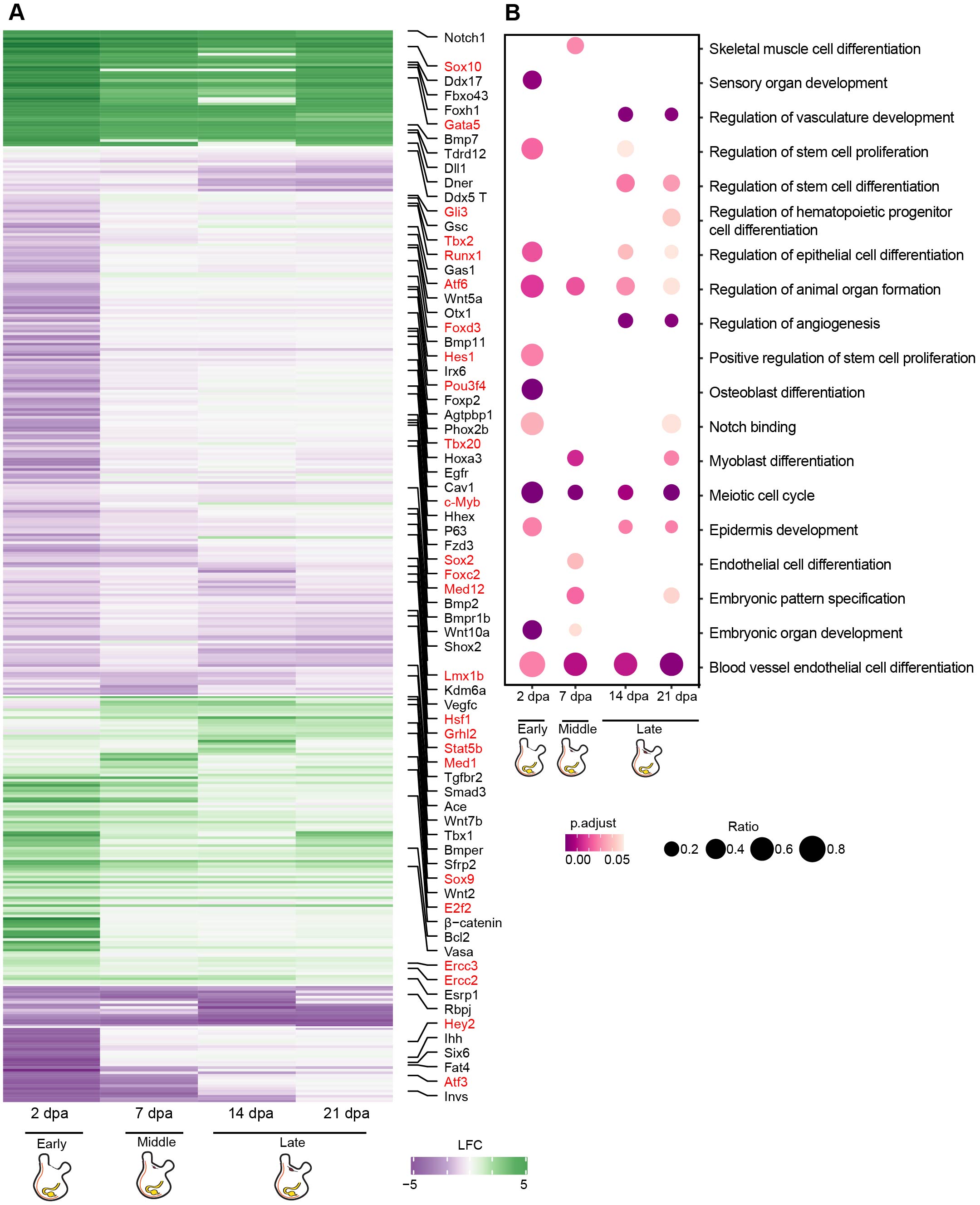

Supplement: Supplementary file 1 [file cells-11-03727-s001.zip › Fig S4.jpg]
